# Prevalence and Associated Factors of Aggressive Behavior among Patients with Schizophrenia at Ayder Comprehensive Specialized Hospital, Ethiopia

**DOI:** 10.1155/2020/7571939

**Published:** 2020-03-23

**Authors:** Tesfalem Araya, Emnet Ebnemelek, Rahel Getachew

**Affiliations:** College of Health Sciences, Mekelle University, Mekelle, Ethiopia

## Abstract

*Introduction*. Mental illness and aggression are often seen as inextricably linked. There is a growing body of evidence on aggressive behavior towards others by people with schizophrenia. Even though there is no study conducted in this study area, this study will help to identify the factors associated with aggressive behavior to be dealt early and as a base line for further study. Therefore, this study was designed to determine the prevalence of aggressive behavior and associated factors among people with schizophrenia.

**Method:**

An institutional-based cross-sectional study was conducted at Ayder Comprehensive Specialized Hospital from May 6 to 31, 2019, among 403 participants who were selected by a systematic random sampling technique. Data was collected by an interview technique by using the Modified Overt Aggression Scale, entered and analyzed by using EPI-INFO version 3.5.3 and Statistical Package for Social Science version 20, respectively. The association between variables was analyzed using bivariate and multivariate logistic regression analyses, and the level of significance of association was determined at a *P* value < 0.05.

**Results:**

A total of 403 schizophrenia patients were included making the response rate 95.4%. The prevalence of aggressive behavior was 26.6%. Significant associated factors for aggressive behavior were being male (AOR = 2.61, 95% CI (1.21, 5.61)), unemployment (AOR = 8.03, 95% CI (3.08, 25.95)), previous history of aggression (AOR = 6.22, 95% CI (2.75, 14.10)), psychotic symptoms (AOR = 8.12, 95% CI (3.11, 21.14)), drug nonadherence (AOR = 6.41, 95% CI (3.02, 13.63)), poor social support (AOR = 3.11, 95% CI (1.35, 7.17)), and alcohol use (AOR = 2.40, 95% CI (1.02, 5.66)).

**Conclusion:**

Prevalence of aggressive behavior is high among schizophrenia patients. Professionals have to identify clearly predictors of aggressive behavior giving special emphasis when treating male schizophrenia patients, who are unemployed, lack social support, with previous history of aggression, and alcohol users.

## 1. Introduction

Mental disorders are leading causes of disability worldwide, accounting for one-third of the years lost due to disability [1]. About 25% of the world's population develops mental illness at some stage in their life [2]. Schizophrenia is one of the serious mental health problems characterized by clinical syndrome of variable, but profoundly disruptive, psychopathology that involves cognition, emotion, perception, and other aspects of behavior [3].

Types of aggression recorded included verbal aggression, aggression towards property, self-harm/autoaggression, and physical aggression [4].

Verbal aggression is typically considered insulting, obscene or profane language, or sexual advances; physical aggression includes hitting, kicking, scratching, pushing, biting, punching, grabbing, pinching, cutting, and stabbing [3]. Aggression against objects includes slamming the door, scattering clothing, throwing objects, kicking or breaking objects, shattering windows, and setting fires. Aggression against self involves picking or scratching the skin, pulling of hair, banging the head or objects, small cuts or bruises, minor burns, mutilating self, deep cuts, and serious injury like suicidal attempt [5].

Schizophrenia has been the diagnosis most often associated with aggression as it has been taken as a paradigm of insanity/psychosis, incompetence, and dangerousness [6].

Patients with serious mental illness (e.g., schizophrenia, major depression, or bipolar disorder) were 2 to 3 times as likely to be assaultive compared with people without such illness [7].

Schizophrenic patients are more likely than those without the disorder to commit violent crimes and aggressive behavior [8].

Aggressive behavior in schizophrenia is estimated to be two to ten times than that of the general population [9].

Although patients suffering from a mental disorder are generally not violent towards others [10], there is a subgroup of potentially dangerous patients with a high risk for violence or aggression [11] and this makes it difficult to reduce the stigma associated with this type of disorder [12]. Certain subgroups of psychiatric patients, including patients who use substance, have psychoses, and are nonadherent to treatment, are aggressive [13]. Patients having more than one category of violence were recorded as having the most aggressive behaviors [14].

Despite this higher prevalence, there is no published data in Ethiopia regarding aggression among schizophrenia patients. Therefore, this study was intended to assess the prevalence of aggression and associated factors among schizophrenia patients who are attending at Ayder Comprehensive Specialized Hospital, Mekelle, Ethiopia.

### 1.1. Prevalence of Aggressive Behavior among People with Schizophrenia

Meta-analysis of published literature indicated that the proportion of aggression in individuals with schizophrenia varies from 6 to 28% [15].

A study done in China shows that the prevalence of aggressive behavior in psychiatric wards ranged between 15.3% and 53.2%. The pooled prevalence of aggression was 35.4% (95% CI: 29.7%, 41.4%) [16].

A study conducted in Canada shows that those with schizophrenia that had the occurrence of aggression over the course of the year was 14.8% [17]. The overall prevalence of violence was 42% for men and 33% for women. Only a weak association was found between gender and violence [18].

A descriptive cross-sectional study done in schizophrenia patients in Spain, using the Modified Overt Aggressive Scale (MOAS), shows 43.95% verbal aggression, 18.68% aggression against objects, 28.57% physical aggression, and 8.8% aggressive against themselves [19].

A study conducted in 2000 in Prague shows that the prevalence of aggression in patients with recurrent schizophrenia was 44.4%. The overall prevalence of aggression was 41.8% for men and 32.7% for women [20]. A study done in the UK shows that those patients with schizophrenia were 41% of the men and 38% of the women and had comorbid diagnoses of abuse and/or dependence [21].

A study conducted in the United States shows that the prevalence of aggressive behavior among schizophrenia patients was 40.9%. Respondents doing something aggressive to others were those who have drug dependence (87.3%) and alcohol dependence (70.9) [22].

A study conducted by the Oregon Psychiatric Association revealed that 42.5% of schizophrenia patients had aggression [23].

Another study conducted in London reported 52% verbal aggression in male patients (46% in females), 39% aggression against objects (25% in females), 23% against self (9% females), and 39% against other people (34% females) as measured by MOAS [24].

A study conducted in Swedish compared the risk of aggressive behavior in patients with schizophrenia; 1054 (13.2%) had at least 1 aggression offense compared with 4276 (5.3%) of the general population controls. The risk was mostly confined to patients with substance abuse comorbidity (of whom 27.6% committed an offense) compared without substance abuse comorbidity; 8.5% of whom had at least one aggressive offense [24].

A study done in Uniting University General Hospital in Madrid shows that 16 (25.4%) were physically aggressive toward others during hospitalization and 9 (14.3%) exhibited threatening behavior [25].

A cross-sectional study conducted in Nigeria, Jos University Teaching Hospital, shows that the prevalence of aggressive behavior was 21.9%.

The patient exhibited 35.15% verbal aggression, 24.20% aggression against property, 9.4% autoaggression, and 31.25% physical aggression with previous acts of aggression 30.6% versus 11.9% at present [26].

Another study conducted in Nigeria, Aro Neuropsychiatric Hospital, shows that the prevalence of aggressive behavior was 23.94 percent [27].

### 1.2. Factors Associated with Aggressive Behavior among People with Schizophrenia

A study conducted in Columbia shows that the risk of aggressive was increasing with (73%) medication noncompliance, (57%) alcohol use, and (51%) previous aggressive behavior. It was significantly associated with being young (under age 40 years) and single, with low social support, residents of urban areas, those who are recently homeless and substance misusers, those with paranoid symptoms, and those with more than two hospital admissions in the prior year [11, 28].

A study conducted in the UK among patients with severe mental illness showed that being male associated with serious assault (41.7%), physical aggression (49.2%), and a life-threatening act of violence against another (21.7%), respectively, while being a woman with serious assault (21.2%), physical aggression (38.8%), and a life-threatening act of violence against another (18.8%), respectively [9].

A study conducted in Denmark and Sweden observed that 30% of all violent offences were committed by males and 50 percent of all violent offences were committed by females. Men are more physically aggressive than women on numerous measures of aggression [29–31].

In a survey of 115 psychiatrists, it was found that 68% of the assaultive patients were 30 years old or younger [32]. Differences in the incidence of disorders between age groups may be a particularly important factor in schizophrenia and drug abuse, in which acute symptoms are more commonly experienced by the young [33]. The risk of future aggression increases with male gender; young adulthood; diagnoses of major mental illness, substance use, persecutory delusions, and command hallucinations; and treatment nonadherence [34].

The Nigerian study shows that aggressive behavior was associated with the age range of the aggressive patients (13 to 66 years), being male and a Christian, and with secondary school level of education and was single or never married, unemployment, and during evening and night periods [27].

A study conducted in Japan indicated that aggressive behavior is associated with unemployment, substance misuse, interpersonal conflict, and crime [35]. Aggression in schizophrenia can be explained by psychopathological symptoms such as delusions or hallucinations, comorbid substance use, social deterioration, or other clinical symptoms [36].

## 2. Methods and Materials

### 2.1. Study Design and Area

An institutional-based cross-sectional study was conducted from May 6 to 31, 2019, at Ayder Comprehensive Specialized Hospital, Mekelle, Tigray, Ethiopia. Ayder Comprehensive Specialized Hospital is one of the biggest teaching and referral hospital in the Tigray region.

### 2.2. Sampling Technique and Sample Size Determination

The sample size was determined using the formula for a single population proportion based on the assumptions for calculating sample size: a 95% confidence level, 5% degree of precision, and 50% is the proportion of aggressive behavior among schizophrenia patient. Then, by adding 10% of the nonresponse rate, the final sample size became 423.The systematic random sampling method was used. The first study participants were selected by a lottery method, and study populations were chosen at regular intervals (every other patient).

### 2.3. Data Collection Instrument

The presence of aggressive behavior among schizophrenia patients was assessed by using the Modified Overt Aggression Scale (MOAS). MOAS has a total of 16 items with four categories that assesses aggression, namely, verbal aggression (with 4 items), aggression against property (with 4 items), autoaggression (with 4 items), and physical aggression (with 4 items). The severity of aggressive behavior ranges from 0 (no aggression) to 4 points (maximum violence) for each category. Each subscale had a weight and multiplied with the respondent's score: verbal aggression weight 1, aggression against property weight 2, autoaggression weight 3, and physical aggression weight 4. Aggressive behavior is defined as a score of 3 or more in any of the MOAS subscores.

Drug adherence was assessed by the 8-item Morisky Medication Adherence Scale (MMAS-8). Scores were categorized as 0-<6 (poor adherence), ≥6-<8 (moderate adherence), and 8 (high adherence). Social support was assessed by using the Oslo-3 Social Support Scale. Its score ranges from 3 to 14, which was then operationalized as “poor support” 3–8, “moderate support” 9–11, and “strong support” 12–14. A semistructured questionnaire was used to collect sociodemographic characteristics and some clinical factors.

### 2.4. Data Collection Tools and Quality Controls

Data was collected by interviewing patients during a routine follow-up visit. The investigator asked the patients whether the patient had behaved aggressively during the past week in any of these domains: verbal aggression, physical aggression towards others or himself/herself, or aggressive behavior towards objects. Training for data collectors was given on how to collect data. A pretest was conducted on 5% of the sample size. Backward and forward translation of the questionnaire was made by a language expert. The collected data was checked on a daily basis for completeness and consistency.

### 2.5. Data Processing and Analysis

The coded data was entered, checked, and cleaned with EPI-INFO version 3.5.3 and analyzed using Statistical Package for the Social Sciences (SPSS) version 20. A descriptive summary using frequencies, percentage, and graphs was used to present study results. Binary logistic regression was done to see the association between outcome variable and explanatory variables. The strength of the association was presented by an odds ratio with 95% confidence interval (CI). A *P* value < 0.05 was considered statistically significant in our study.

## 3. Results

Four hundred three respondents participated in the study making a response rate of 95.3%.

### 3.1. Sociodemographic Characteristics

A total of 403 schizophrenia patients were included in this study; 266 (66.0%) were male, and the mean age of participants was 33.9 years (SD ± 10.28) ranged from 18 to 60 (Table 1).

### 3.2. Clinical Factors

Concerning clinical factors of the participants, 222 (55.1%) had the diagnosis of recurrent schizophrenia, while 300 (74.4%) were on conventional antipsychotic drug (Table 2).

**Figure 1:**
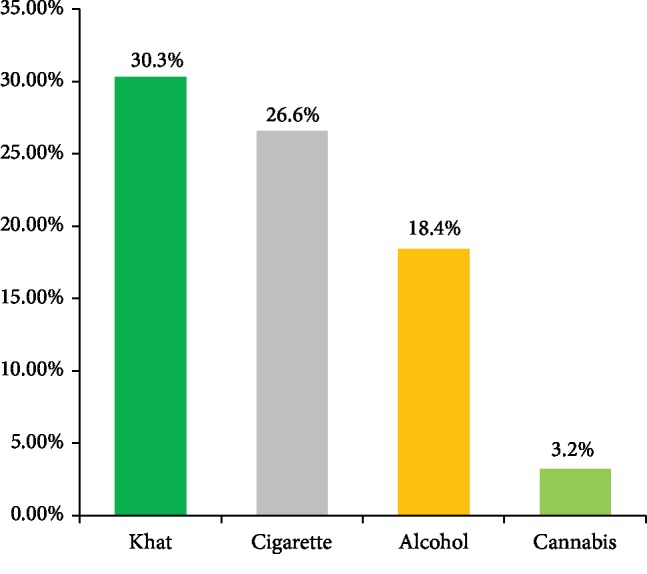
The type of current substance used among people with schizophrenia attending Ayder Comprehensive Specialized Hospital since aggression initiation, May, 2019.

Regarding the current substance use among schizophrenia patients, 316 (78.5%) participants used substance, with 122 (30.3%) using khat (Figure 1).

### 3.3. Prevalence of Aggressive Behavior

**Figure 2:**
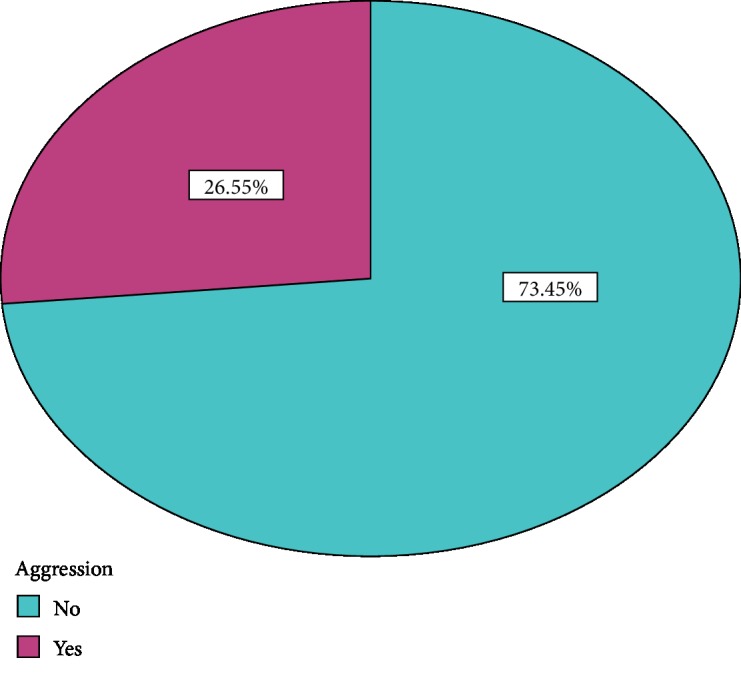
Prevalence of aggressive behavior among people with schizophrenia, attending Ayder Comprehensive Specialized Hospital, May, 2019.

The overall prevalence of aggression among schizophrenia patients in our study was found to be 26.6% [24.2-29.7] (Figure 2).

**Figure 3:**
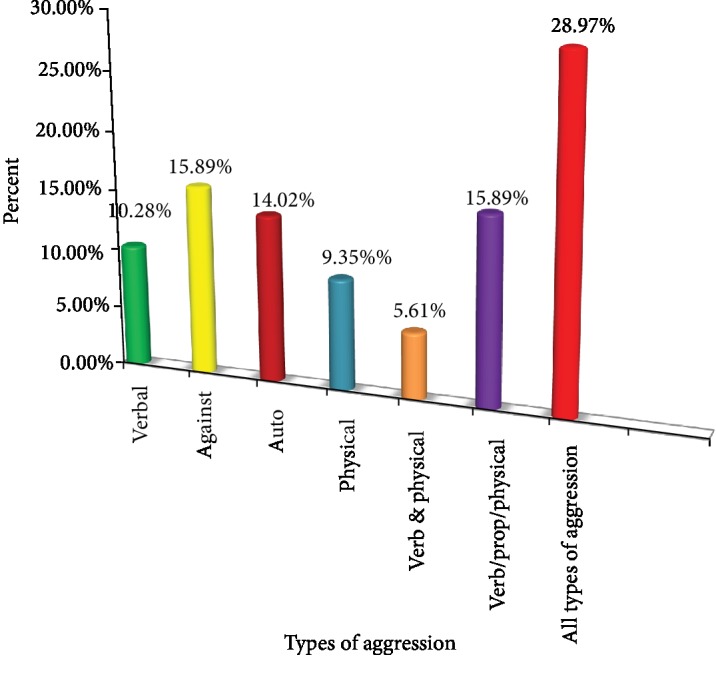
Subtypes of aggressive behavior among people with schizophrenia attending Ayder Comprehensive Specialized Hospital, May, 2019.

Of the total respondents, 11 (10.28%) have verbal aggression, 17 (15.89%) have aggression against property, 15 (14.02%) have autoaggression, 10 (9.35%) have physical aggression, 6 (5.61%) have both verbal and physical aggression, 17 (15.89%) have verbal and against property and physical aggression, and 31 (28.97%) have all types of aggression (Figure 3).

### 3.4. Bivariate Analysis

On bivariate logistic regression, sex, monthly income, marital status, occupation, diagnosis of schizophrenia, previous history of aggression, types of medication, current psychotic symptom, drug nonadherence, social support, alcohol use, khat use, and cannabis use were statistically significant at crude odds ratio and entered in to multivariate binary logistic regression analysis.

### 3.5. Multivariate Analysis

During the multivariate analysis, sex, occupation, previous history of aggression, current psychotic symptom, drug nonadherence, social support, and alcohol use were found to be statistically significant.

Male patients were more than two times more likely to be aggressive as compared to female patients with the odds of (AOR = 2.61, 95% CI (1.21, 5.61)). Patients who had no job were about eight times more likely to be aggressive as compared with patients who had a job with the odds of (AOR = 8.03, 95% CI (3.08, 25.95)) (Table 3).

## 4. Discussion

In our study, the overall prevalence of aggressive behavior among schizophrenia patients was higher than that in the study done in Canada (14.8%) [17], Nigeria (21.9%) [26], and Aro Neuropsychiatric Hospital (23.94%) [27], respectively. Meta-analysis of published literature indicated that the prevalence of aggressive behavior in schizophrenia patients ranged from 6% to 28% [15] and was lower than the study done in China which ranged between 15.3% and 53.2% with pooled prevalence of aggression 35.4% (95% CI: 29.7%, 41.4%) [16]. The possible reason for the difference in the magnitude of prevalence can be due to the sociocultural difference in the study population, clinical-related factors, and methodological differences.

About 55.3% of study participants in the current study area had a recurrent episode of schizophrenia which was higher than that in the study done in Prague 44.14% and in Nigeria 24.6% [27]. This difference could be due to higher substance use in this study area (78.41%) and Canada 34% [17].

Domains of aggressive behavior in current study area revealed that 11 (10.28%) had verbal aggression; 17 (15.89%) aggression against property; 15 (14.02%) autoaggression; 10 (9.35%) physical aggression; 6 (5.61%) had both verbal and physical aggression; 17 (15.89%) had verbal aggression, aggression against property, and physical aggression; and 31 (28.97%) have had all types of aggression.

The most common type of aggressiveness was against property and the least common was physical which is different with the study done in Spain and London [19, 24]. The possible reason for aggression may be partly explained socioculturally in the sense that in our environment, aggression against property or physical exchange is often a means of settling conflicts instead of discussing issues. Most of the time, aggression was observed in the evening and night time 16.1% which was in line with the study done in Nigeria [27].

After multivariate binary logistic regression analysis, being male increases the risk of aggression by more than two times than being female (AOR = 2.61, 95% CI (1.21, 5.61)), which is in line with a study done in Nigeria and the UK [9, 27].

Patients who were jobless have an increased risk of aggression eight times than being employed (AOR = 8.03, 95% CI (3.08, 21.95)), which is in line with study done in Nigeria and Japan [27, 35].

Patients who had a previous history of aggression were about six times more likely to be aggressive when compared to patients who had no history of aggression (AOR = 6.22, 95% CI (2.75, 14.10)) which is in line with the study done in Nigeria [27].

Patients who had a current psychotic symptom among study participants and those who had commanding hallucination and persecutory delusion were about eight and seven times more likely to be aggressive as compared to patients who had no psychotic symptoms (AOR = 8.12, 95% CI (3.11, 21.14)) and (AOR = 7.85, 95% CI (2.88, 21.39)), respectively, which is in line with the study done in Nigeria, Canada, and Columbia [11, 17, 26].

The possible reason could be that aggression is known to occur in response to psychotic experience, especially delusions and hallucinations.

Concerning drug adherence, those who have nonadherence to prescribed medications were about six times more likely to be aggressive than those who adhere to their medication (AOR = 6.41, 95% CI (3.02, 13.63)) which is in line with the study done in Columbia [11]. Stopping or terminating antipsychotic medication was the main factor for relapsing and aggressive behavior.

Patients who had poor social support were three times more likely to be aggressive as compared to patients who had strong social support (AOR = 3.11, 95% CI (1.35,7.17)) which is in line with the study done in Columbia and Japan [11, 35]. Poor social support decreases medication adherence which in turn increases aggression behavior in schizophrenia.

Concerning substance use, patients who were drinking alcohol before aggression were found to be two times more likely aggressive than patients who had no history of alcohol use (AOR = 2.40, 95% CI (1.02, 5.66)) which is in line with study done in the USA and Columbia [11, 22]. Substance use increases the aggression and restlessness of schizophrenia patients. The possible reason may be the use of these substances had an influence on the effect of medication adherence and aggravate active symptoms.

## 5. Conclusion

Prevalence of aggressive behavior is high among schizophrenia patients who are attending at Ayder Comprehensive Specialized Hospital outpatient clinic. Being male, jobless, history of past aggression, presence of psychotic symptoms, medication nonadherence, poor social support, and current alcohol use were significantly associated with aggression. Clinicians have to identify reliable clinical and illness predictors for aggression in schizophrenia patients aimed at reducing aggressive behaviors and their adverse outcomes.

### 5.1. Limitation

This study was a cross-sectional study design that cannot show the temporal cause-effect association between factors and aggressive behavior. This study did not measure the severity of psychotic symptoms that elicited aggressive behavior but rather the emphasis on types of psychotic symptoms that preceded an act of aggression.

## Figures and Tables

**Table 1:** Demographic characteristics of study subjects (*N* = 403).

Variables	*N* (%)
Age	
18-24	65 (16.1)
25-34	165 (40.9)
35 -44	111 (27.5)
45-54	39 (9.8)
≥55	23 (5.7)
Sex	
Male	266 (66)
Female	137 (34)
Residence	
Urban	230 (57.1)
Rural	173 (42.9)
Income	
<400 birr	169 (41.9)
400-900	57 (14.1)
900-1200	99 (24.6)
>1200	78 (19.4)
Religion	
Orthodox	215 (53.3)
Muslim	133 (33.1)
Protestant	55 (13.6)
Marital status	
Single	198 (49.1)
Separated	68 (16.9)
Divorced	51 (12.7)
Married	86 (21.3)
Ethnicity	
Tigray	217 (53.84)
Amhara	75 (18.64)
Afar	65 (16.12)
^∗^Other	46 (11.40)
Education	
No formal education	120 (29.8)
Primary	109 (27.0)
Secondary	116 (28.8)
Diploma and above	58 (14.4)
Occupation	
Employed	62 (15.4)
Own business	122 (30.3)
Student	29 (7.2)
Jobless	190 (47.1)
Living circumstance	
With family	350 (86.8)
Alone	53 (13.2)

^∗^Other: Gurage, Welayita, Sidama, and Oromo.

**Table 2:** Clinical characteristics of study subjects in relation to aggressive behavior (*N* = 403).

Variables	*N* (%)
Diagnosis	
First episode	40 (9.9)
Second episode	141 (35.0)
Recurrent	222 (55.1)
History of aggression	
Yes	70 (17.4)
No	333 (82.6)
Types of drug taken	
Typical antipsychotic	300 (74.4)
Atypical antipsychotic	103 (25.6)
Nature of aggression	
Verbal aggression	11 (10.28)
Aggression against objects	17 (15.89)
Autoaggression	15 (14.02)
Physical aggression	10 (9.35)
Both verbal & physical	6 (5.61)
Verbal/prop/physical	17 (15.89)
All types of aggression	31 (28.97)
Time of aggression	
None	263 (65.3)
Morning	35 (8.7)
Afternoon	40 (9.9)
Evening/night	65 (16.1)
Current symptoms	
No symptoms	297 (74.7)
Persecutory delusion	52 (13.2)
Command hallucination	54 (12.1)
Drug adherence	
Nonadherence	208 (51.6)
Adherence	195 (48.4)
Social support	
Poor support	163 (40.4)
Moderate support	157 (39.0)
Strong support	83 (19.6)
Current substance use	
Yes	316 (78.5)
No	87 (21.5)

**Table 3:** Distribution of schizophrenia patients by factors related to aggressive behavior at Ayder Comprehensive Specialized Hospital, May, 2019 (*n* = 403).

Explanatory variables	Aggressive behavior		COR (95% CI)	AOR (95% CI)
	Yes	No		
Sex				
Male	80	186	1.75 (1.07, 2.88)^∗^	2.61 (1.21, 5.61)^∗∗^
Female	27	110	1.00	1.00
Monthly income				
<400 birr	57	112	2.14 (1.12, 4.08)^∗^	1.32 (0.53, 3.26)
400-900 birr	22	35	2.64 (1.22, 5.73)^∗^	3.28 (.70, 10.04)
900-1200 birr	13	86	0.63 (0.28, 0.99)^∗^	0.64 (0.20, 2.01)
>1200 birr	15	63	1.00	1.00
Occupational status				
Employed	7	55	1.00	1.00
Own business	7	115	0.48 (0.16, 0.93)^∗^	0.25 (0.65, 0.99)
Students	9	20	3.54 (1.16, 10.75)^∗^	5.28 (0.18, 23.52)
Jobless	84	106	6.23 (2.69, 14.38)^∗^	8.03 (3.08, 21.95)^∗∗^
History of aggression				
Yes	40	30	5.29 (3.07, 9.12)^∗^	6.22 (2.75, 14.10)^∗∗^
No	60	266	1.00	1.00
Psychotic symptoms				
No symptoms	55	242	1.00	1.00
Persecutory delusion	24	28	3.77 (2.03, 7.00)^∗^	7.05 (2.88, 21.39)^∗∗^
Command hallucination	28	26	4.74 (2.57, 8.71)^∗^	8.12 (3.11, 21.14)^∗∗^
Drug adherence				
Nonadherence	84	124	5.07 (3.02, 8.48)^∗^	6.41 (3.02, 13.63)^∗∗^
Adherence	23	172	1.00	1.00
Social support				
Poor social support	70	93	2.72 (1.48, 4.98)^∗^	3.11 (1.35, 7.17)^∗∗^
Moderate support	19	138	0.49 (0.25, 0.99)^∗^	0.56 (0.22, 1.43)
Strong support	18	65	1.00	1.00
Alcohol				
Yes	27	44	1.93(1.13, 3.32)^∗^	2.40(1.02, 5.66) ^∗∗^
No	80	332	1.00	1.00

Key: ^∗^*P* value < 0.05; ^∗∗^Statistically significant at multivariate analysis.

## Data Availability

Data generated or analyzed during this study are included in this published paper. The datasets during the current study was supplied from the corresponding author on reasonable request.
